# Impaired High-Density Lipoprotein Anti-Oxidant Function Predicts Poor Outcome in Critically Ill Patients

**DOI:** 10.1371/journal.pone.0151706

**Published:** 2016-03-15

**Authors:** Lore Schrutka, Georg Goliasch, Brigitte Meyer, Raphael Wurm, Lorenz Koller, Lukas Kriechbaumer, Gottfried Heinz, Richard Pacher, Irene M Lang, Klaus Distelmaier, Martin Hülsmann

**Affiliations:** 1 Department of Internal Medicine II, Medical University of Vienna, Vienna, Austria; 2 Department of Internal Medicine IV, Kaiser-Franz-Josef-Hospital Vienna, Vienna, Austria; 3 University Clinic of Orthopedics, Paracelsus Medical University Salzburg, Salzburg, Austria; Kermanshah University of Medical Sciences, ISLAMIC REPUBLIC OF IRAN

## Abstract

**Introduction:**

Oxidative stress affects clinical outcome in critically ill patients. Although high-density lipoprotein (HDL) particles generally possess anti-oxidant capacities, deleterious properties of HDL have been described in acutely ill patients. The impact of anti-oxidant HDL capacities on clinical outcome in critically ill patients is unknown. We therefore analyzed the predictive value of anti-oxidant HDL function on mortality in an unselected cohort of critically ill patients.

**Method:**

We prospectively enrolled 270 consecutive patients admitted to a university-affiliated intensive care unit (ICU) and determined anti-oxidant HDL function using the HDL oxidant index (HOI). Based on their HOI, the study population was stratified into patients with impaired anti-oxidant HDL function and the residual study population.

**Results:**

During a median follow-up time of 9.8 years (IQR: 9.2 to 10.0), 69% of patients died. Cox regression analysis revealed a significant and independent association between impaired anti-oxidant HDL function and short-term mortality with an adjusted HR of 1.65 (95% CI 1.22–2.24; p = 0.001) as well as 10-year mortality with an adj. HR of 1.19 (95% CI 1.02–1.40; p = 0.032) when compared to the residual study population. Anti-oxidant HDL function correlated with the amount of oxidative stress as determined by Cu/Zn superoxide dismutase (r = 0.38; p<0.001).

**Conclusion:**

Impaired anti-oxidant HDL function represents a strong and independent predictor of 30-day mortality as well as long-term mortality in critically ill patients.

## Introduction

Despite intensive research and significant advances in the management of critical ill patients over the last decades, prognosis remains poor [1,2]. Oxidative stress, resulting from an imbalance between reactive oxygen species (ROS) production and anti-oxidant defenses, is known to seriously affect clinical outcome by exacerbating organ damage [3]. Potential sources of increased ROS generation in this highly vulnerable patient population are activated immune cells, the vascular endothelium, the release of iron and copper ions and metalloproteinases, as well as ischaemia/reperfusion-induced tissue injury [3,4]. Increased ROS production during critical illness is accompanied by a disturbance of anti-oxidant systems leading to a severe accumulation of free radicals [4]. This overwhelming oxidative stress response leads to cellular damage through a number of pathways including direct damage to proteins, membranes, DNA and RNA or indirect damage through the activation of pro-inflammatory and pro-apoptotic pathways [4–6]. Consequently, an optimization of anti-oxidant defense is regarded as promising therapeutic approach in patients admitted to the intensive care unit (ICU) [7,8,9].

High-density lipoprotein (HDL) particles possess strong anti-oxidant properties by binding and removing oxidant molecules [10]. Thus HDL is believed to play an important role in scavenging oxidative stress in patients with high levels of oxidative stress. However, there is rising evidence that inflammation and tissue injury, can significantly compromise the protective properties of HDL [11,12]. HDL particles may even amplify pro-oxidant processes in clinical conditions associated with inflammation and oxidative stress [13,14]. A recently published study revealed for the first time a strong association between preserved anti-oxidant capacities of HDL and survival in patients with acute myocardial infarction [14]. This finding is in line with previous studies suggesting that HDL function may relate more to clinical outcome than quantitative serum levels [11,15].

The quality of HDL has not been analyzed in critical ill patients yet. Previous analyses revealed a substantial reduction of HDL serum levels in critical ill patients [16,17]. In view of the underlying inflammatory processes [3,4], it is tempting to speculate that HDL quality is significantly impaired in this highly vulnerable patient population. We therefore analyzed the anti-oxidant capacity of HDL in an unselected cohort of patients admitted to the ICU with a simple serum-based assay, and determined its association with survival.

## Materials and Methods

### Study Population

We prospectively enrolled all patients admitted to the intensive care unit (ICU) of the Department of Cardiology of the Vienna General Hospital, a university-affiliated tertiary care center, between August 2004 and November 2005 in our study as previously described [18]. Patients aged <18 years were excluded. The study protocol complies with the Declaration of Helsinki and was approved by the Ethics Committee of the Medical University of Vienna; written informed consent was obtained from the patient, patient relatives or the appointed legal guardian.

Baseline characteristics and clinical history were recorded in all patients. According to their primary diagnosis patients were divided into cardiac or non-cardiac patients and further subdivided into postoperative or internal reasons of admission. A non-cardiac patient was defined with a primary non-cardiac diagnosis (e.g. pneumonia) and did not preclude a secondary cardiac disease, nor was a preexisting cardiac disease a priori excluded. All major interventions prior to ICU admittance as well as within the first 48 hours of ICU stay were recorded, as were presence of renal failure and need for extracorporeal renal replacement therapy, mechanical ventilation, presence of shock and shock category, support therapy with extracorporeal membrane oxygenation, use of intra-aortic balloon counterpulsation, resuscitation, presence of left ventricular dysfunction and heart surgery. Severity of disease was quantified by the Simplified Acute Physiology Score (SAPS) II [19]. Extracorporeal therapy was defined as renal replacement therapy as well as extracorporeal membrane oxygenation at the time of blood sample collection. All-cause 30-day mortality was defined as primary and long-term mortality during the whole observation period as secondary study endpoint. Mortality data were obtained by screening of the national register of death.

### Laboratory measurements

Routine laboratory assessment was performed in all patients at time of admission according to the local laboratory’s standard procedure. Further blood samples were collected for determination of anti-oxidant HDL-function and measurement of Cu/Zn superoxide dismutase within 24 hours of ICU admission as well as 48h after ICU admission. All samples were stored at -80°C and used for further analyses immediately after thawing.

HDL anti-oxidant capacity was measured using a 2’,7’-dichlorodihydrofluorescein diacetate (DCF)-based cell free fluorescent assay that determined the ability of apolipoprotein (apo)-B depleted serum, which includes HDL, apo-A1, apo-A2, and HDL associated particles, to inactivate or aggravate previously oxidized low-density lipoprotein (LDL) [20]. The assay was performed as previously described [14]. Briefly, LDL (Merck, Milipore, Darmstadt, Germany) was diluted in phosphate buffered saline (PBS) to a final cholesterol end-concentration of 100μg/ml and oxidized for 6 hours in 100μM CuSO4 (Merck) at 37°C by dialysis. After HDL containing supernatant was obtained from the patient serum samples by polyethylene glycol depletion (15μl), DCF-DA (final concentration 2.9μg/ml) as well as oxidized LDL (final concentration: 1.4μg/ml) were added to separate wells of 96-well black microplates and incubated at 37°C with PBS to a final volume of 175μl in 96-well plates with clear bottom for one hour (Corning, Amsterdam, Netherlands). The fluorescence signal was measured at an excitation of 485nm and emission wavelength of 530nm using a Synergy H1 Hybrid Microplate Reader (Biotek, Winooski, VT, USA). All patient samples were run in duplicate and a pooled serum control from 3 healthy volunteers was added on each plate. Inter-assay coefficient of variation was 7.8%, whereas intra-assay coefficient of variation was 5.9%. To calculate the HDL oxidant index (HOI), the intensity signal of DCF-fluorescence alone was subtracted from the intensity signal of DCF incubated with apolipoprotein B–depleted patient sera and log-transformed before analysis [13,21]. Accordingly, the higher the HOI the poorer the anti-oxidant function. To correct for inter-assay variability across different plates, a pooled serum control from 3 healthy volunteers was added on each plate, and values for samples from subjects in the study were normalized by this pooled value. Additionally, plasma levels of Cu/Zn superoxide dismutase, a marker for oxidative stress, independent from diabetes mellitus and inflammation [22] were determined using a commercially available ELISA kit (Bender MedSystems, Vienna, Austria. All other laboratory parameters were analyzed according to the local laboratory’s standard procedure at time of study enrollment.

### Statistical analyses

Discrete data were described by absolute and relative frequencies and compared between groups using Chi-square tests. Because of the asymmetrical distribution, continuous data were presented as medians with interquartile ranges (IQR) and compared between groups using the Mann-Whitney U-tests. Wilcoxon signed-rank tests were applied for pairwise comparison in subgroup analysis. Spearman correlation was performed to assess the relationship between continuous variables. HOI was stratified into tertiles and the third tertile of HOI has been chosen as cut-off to divide study population into patients with impaired antioxidant HDL function (high HOI) and residual study population. The influence of having high HOI on 30-day mortality as well as long-term mortality was investigated by univariate and multivariable Cox regression models. Multivariate adjustment for potential clinical confounders included the Simplified Acute Physiology Score (SAPS) II [19], age, sex, extracorporeal therapy, cholesterol levels, glomerular filtration rate (GFR) and the use of catecholamines. Kaplan-Meier analyses were applied to measure the effect of HOI on 30-day mortality and long-term mortality and compared using log-rank test. The discriminatory power of the respective variables was assessed using ROC analysis. Two-sided p-values less than 0.05 were used to indicate statistical significance. SPSS 21.0 (SPSS/IBM) was used for all analyses.

## Results

### Baseline characteristics

A total of 289 patients admitted to the ICU were consecutively enrolled during the initial study period [18]. From 19 patients, no frozen serum samples for determination of anti-oxidant HDL function were available, leaving 270 patients for the final analysis. Sixty-nine percent of patients (n = 187) were male and the median age was 65 years (IQR 55–75). Thirty percent of the study population was post-operatively admitted to the ICU and 67% was hospitalized due to cardiac reasons. Primary diagnoses are summarized in *Table 1*. The median SAPS-2 score of the study population was 55 (IQR 41–73). Within the first 48 hours of ICU admission, a total of 208 patients (62%) were on catecholamines, 220 patients (82%) required mechanical ventilation, 66 patients (24%) developed acute renal failure, and 29 patients (11%) required renal replacement therapy. Twenty-one patients were treated with intra-aortic balloon counter pulsation, eight patients were on extracorporeal membrane oxygenation support, and three patients had left ventricular or biventricular assist devices. Forty-three patients (16%) were resuscitated before or within 48 hours after ICU admission. According to the baseline HOI, the study population was stratified into patients with impaired anti-oxidant function of HDL, characterized by a high HOI (HOI>5.90) and residual study population (HOI≤5.90). Detailed baseline characteristics and characteristics for patients with impaired anti-oxidant HDL function and the residual study population are displayed in *Table 2*. During a median follow-up time of 9.8 years (IQR 9.2–10.0), 69% of patients (n = 185) died. Causes of death were cardiovascular disease (65%), malignancy (14%), lung disease (7%) metabolic disorders (4%), gastro-intestinal disease (4%) and other causes (7%).

**Table 1:** 

Primary Diagnosis		No.
Cardiac		
Myocardial infarction		28
Cardiogenic shock		20
Heart failure		19
Pulmonary edema		8
Pericardial effusion		7
Arrhythmia		13
Valvular heart disease		8
Coronary artery bypass surgery		27
Valve surgery		17
Bypass and valve surgery		9
Cardiac surgery, other		3
Aortic aneurysm		5
Artificial heart		3
Pulmonary hypertension		2
Endocarditis		2
Resuscitation		24
Transplantation		
Heart		4
Lung		2
Infectious		
Sepsis		7
Neurologic		
Stroke		3
Meningitis		1
Cerebral bleeding		2
Seizure		1
Coma		2
Brain death		2
Respiratory		
COPD		11
Respiratory failure		14
Pneumonia		9
Pulmonary embolism		6
Lung Bleeding		1
Oncologic		
Esophageal cancer		1
Bronchus carcinoma		1
Bleeding		5
Cirrhosis hepatis		1
Intoxication		1
Hypothermia		1

**Table 2:** Baseline characteristics of total study population and for patients with impaired anti-oxidant HDL function (HDL oxidant index (HOI)>5.90) and the residual study population (HOI≤5.90).

	Total study population	HOI≤5.90	HOI>5.90	*P* value
N	270	180	90	
HOI (IQR)	5.14 (4.06–6.80)	4.35 (3.72–5.14)	8.01 (6.79–9.91)	**<0.001**
Age, median years (IQR)	65 (55–75)	64 (55–75)	67 (58–75)	0.388
Male sex, n (%)	187 (69)	131 (73)	56 (62)	0.076
Body weight, kg (IQR)	80 (70–90)	80 (70–90)	80 (70–91)	0.988
MAP, mmHG (IQR)	58 (49–67)	59 (53–68)	57 (45–66)	**0.018**
Heart rate, bpm (IQR)	94 (57–118)	91 (55–115)	99 (66–120)	0.210
Cholesterol, mg/dl (IQR)	150 (119–188)	150 (117–190)	153 (126–185)	0.432
HDL-C, mg/dl (IQR)	37 (31–48)	37 (29–47)	42 (33–52)	0.151
LDL-C, mg/dl (IQR)	100.20 (71.10–130.10)	97.20 (66.90–130.10)	101.60 (79.20–136.40)	0.509
Cholesterol/HDL quotient (%)	4.3 (3.4–5.7)	4.3 (3.4–5.9)	4.5 (3.4–5.3)	0.743
Triglycerides, mg/dl (IQR)	120 (89–175)	115 (88–169)	128 (89–189)	0.256
Leukocytes, 10^9^/L (IQR)	11.71 (8.48–15.37)	11.70 (8.61–15.08)	12.10 (8.17–16.63)	0.652
Neutrophiles, 10^9^/L (IQR)	8.60 (6.00–11.10)	8.65 (6.02–11.47)	8.10 (5.70–10.50)	0.446
Cu/Zn SOD, nmol/l (IQR)	9.65 (6.26–15.32)	8.05 (5.87–12.43)	13.85 (8.58–18.35)	**<0.001**
Creatinine, mg/dl (IQR)	1.3 (0.9–1.9)	1.2 (0.9–1.7)	1.4 (1.0–2.0)	**0.011**
ASAT, U/L (IQR)	62 (36–158)	62 (36–148)	66 (34–210)	0.543
ALAT, U/L (IQR)	37 (18–90)	37 (18–88)	36 (20–99)	0.937
γ-GT, U/L (IQR)	56 (29–114)	56 (30–112)	57 (27–121)	0.816
CRP, mg/dl (IQR)	3.9 (0.8–12.7)	3.8 (0.7–11.1)	3.9 (1.0–17.5)	0.238
Estimated GFR, ml/min (IQR)	59.67 (39.41–85.31)	64.12 (40.21–95.74)	53.37 (33.50–74.57)	**0.019**
Catecholamines, n (%)	208 (77)	135 (75)	73 (81)	**0.154**
SAPS II, points (IQR)	55 (41–73)	53 (40–66)	64 (45–80)	**0.001**
Extracorporeal circulation, n (%)	35 (13)	17 (9)	18 (20)	**0.015**
Extracorporeal membrane oxygenation, n (%)	8 (3)	3 (1)	5 (6)	0.076
Haemodiafiltration, n (%)	29 (11)	14 (8)	15 (17)	**0.028**
Postoperative admission to ICU, n (%)	80 (30)	51 (28)	29 (32)	0.510
Cardiac admission, n (%)	181 (67)	123 (68)	58 (64)	0.522
Length of ICU stay, days (IQR)	6 (3–12)	6.5 (4–12)	4.5 (2–13)	0.052

### Association of HDL function with clinical risk factors

Impaired anti-oxidant function of HDL was found significantly associated with higher levels of Cu/Zn superoxide dismutase, a marker of oxidative stress (r = 0.38; p<0.001). Furthermore, HOI showed an inverse correlation with the GFR (r = -0.17, p = 0.009). Further, sub-analysis revealed a significantly higher HOI in individuals undergoing extracorporeal circulation at the time of blood sample collection (5.93 [IQR 4.96–8.06] vs. 4.88 [IQR 4.02–6.63], p = 0.008). No differences in HOI, determined 48h after ICU admission, were observed between surgical (5.05 [IQR 4.14–7.18]) and non-surgical ICU patients (5.15 [IQR 4.06–6.62] p = 0.577), between patients admitted for cardiac (4.93 [IQR 4.05–6.78]) and non-cardiac reasons (5.30 [IQR 4.23–6.79] p = 0.401) as well as between men (5.03 [IQR 3.97–6.53]) and women (5.46 [IQR 4.30–7.25] p = 0.066). HOI was independent of serum HDL-cholesterol levels (r = 0.017, p = 0.848).

To analyze the changes of anti-oxidant HDL function during the ICU stay, we compared the HOI between serum samples collected on the first day of ICU admission and 48 hours after ICU admission. HOI was found significantly higher on the first day of ICU admission (5.72 [IQR 4.24–8.75] vs. 5.14 [IQR 4.06–6.79], p<0.001) demonstrating an improvement of anti-oxidant HDL function within the first 48 hours after ICU admission. Sub-analyses revealed that improvement of anti-oxidant HDL function within the first 48 hours after ICU admission was only observed in surgical patients (9.26 [IQR 5.24–13.14] vs. 5.11 [IQR 4.24–7.19], p<0.001), whereas in non-surgical patients a stable HOI was observed (5.35 [IQR 4.04–7.43] vs. 5.14 [IQR 4.03–6.62], p = 0.104).

### Survival analyses

**Fig 1:**
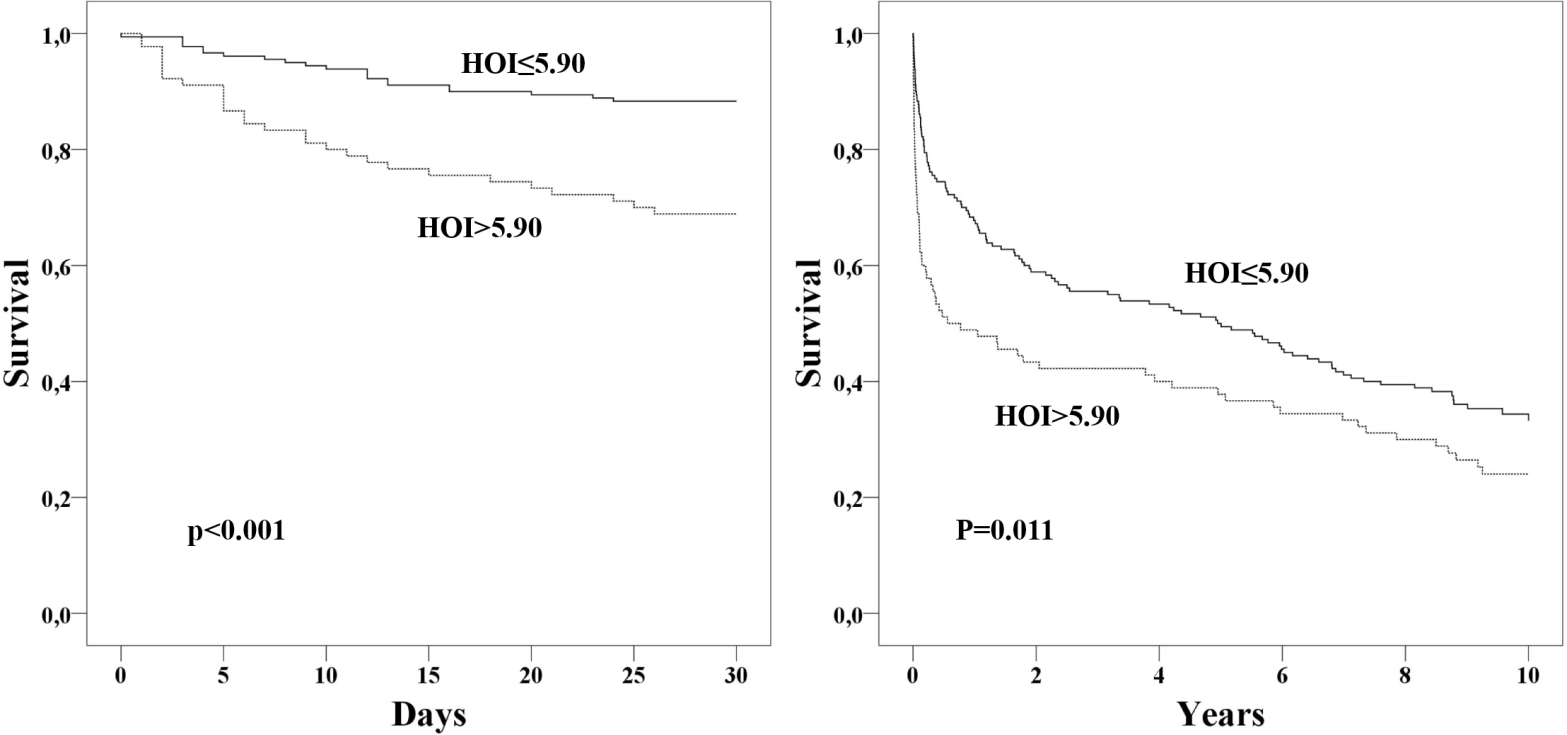
Kaplan-Meier plots showing 30-day mortality A) and 10-year mortality B) for critically ill patients with impaired anti-oxidant HDL function [high-density lipoprotein inflammatory index (HOI>5.90)] and the residual study population (HOI≤5.90). Indicated p-values were derived from log rank test.

We identified a significant association between high HOI and 30-day mortality with a crude hazard ratio (HR) of 1.73 (95% CI 1.31–2.30; p<0.001) as well as 10-year long-term mortality with a crude HR of 1.21 (95% CI 1.04–1.41; p = 0.012) when comparing patients with the residual study population. This effect persisted after adjustment for potential confounders with an adjusted HR of 1.65 (95% CI 1.22–2.24; p = 0.001) for 30-day mortality and an adjusted HR of 1.19 (95% CI 1.02–1.40; p = 0.032) for 10-year long-term mortality. The area under the ROC curve (AUC) for HOI was 0.66 for 30-day mortality and 0.55 for 10-year long-term mortality. Kaplan-Meier analysis revealed a significant increase of 30-day mortality (p<0.001, log-rank test; Fig 1A) and long-term mortality (p = 0.011, log-rank test; Fig 1B) in patients with high HOI. Survival was decreased in patients with high HOI after 30 days (31% vs. 12%) and after 10 years (76% vs. 65%) compared to the residual study population.

## Discussion

In the present study, we analyzed for the first time the protective properties of HDL in critically ill patients and identified a strong association between anti-oxidant HDL function and clinical outcome. Short-term as well as 10-year long-term survival was significantly reduced in patients with impaired anti-oxidant HDL function reflected by a high HOI. These associations were even more pronounced after adjustment for potential confounders. Anti-oxidant HDL function, determined 48hours after ICU admission, was selected as risk predictor in order to await the initial massive stress response associated with the ICU admission [23].

So far, the role of HDL was not in the focus of previous research in ICU patients. Although a reduction of HDL serum levels has been described in critical ill patients [16,17], the quality of HDL has never been investigated. This lack of HDL research in ICU patients is primarily caused by the former assumption that the main function of HDL is restricted to the reverse cholesterol transport. However, over the last years it has become evident that HDL particles possess anti-oxidant, anti-inflammatory, anti-thrombotic and anti-apoptotic properties besides reverse cholesterol transport [24]. Considering the pivotal role of oxidative stress and inflammation during critical illness [3,4], these properties of HDL may significantly affect clinical outcome. Consequently, HDL has gained more attention in the acute clinical setting over the last years [5,13,14]. In patients with acute myocardial infarction significantly impaired HDL function have been found [13,14,25] strongly associated with increased mortality [14]. Our results are the first to demonstrate an association between anti-oxidant HDL function and clinical outcome in a heterogeneous population of acutely ill patients, including surgical and non-surgical patients.

There exists strong evidence that oxidative stress dramatically increases during critical illness, specifically in patients with sepsis, cardiovascular disorders, cardiogenic shock, organ dysfunction, acute respiratory distress syndrome, acute renal failure, and disseminated intravascular coagulation [26,27,28]. Activated phagocytic cells, the vascular endothelium, and ischaemia/reperfusion-induced tissue injury have been identified as potential sources of oxidative stress. Oxidative stress results in damage to proteins, membranes, DNA and RNA [4] and has been found associated with severity and progress of disease in critical ill patients [28,29]. An increase of ROS levels were found accompanied by a decrease in anti-oxidant defenses [30]. Consequently, the prevention and control of ROS production is suggested to be a potential therapeutic target in ICU patients.

The present study revealed a strong association between survival and anti-oxidant HDL function in a highly vulnerable ICU patient population. In patients with poor anti-oxidant HDL function, higher levels of oxidative stress were measured. Furthermore, patients with impaired anti-oxidant HDL function showed a trend towards longer ICU stays. These findings underline the essential role of HDL in the anti-oxidant defense. The underlying pathophysiological mechanisms for impaired anti-oxidant function of HDL have not been fully elucidated yet but there is strong evidence that changes in HDL protein composition and protein quality are crucially involved [31]. These structural changes of HDL particles may be provoked by an acute stress response during conditions of infection, inflammation or tissue injury [32]. Furthermore, oxidative stress itself is known to trigger post-translational modifications of HDL-associated proteins that may render HDL dysfunctional [32,33]. Particularly, myeloperoxidase (MPO), which is mainly secreted by activated neutrophils, has been identified to affect HDL function by promoting oxidative damage on HDL associated proteins [33–35]. Similarly, replacement of apolipoprotein A1 by serum amyloid A on the surface of the HDL particle and altered enzymatic activities, including paraoxonase-1 and platelet-activating factor acetylhydrolase, might contribute to the impairment of anti-oxidant HDL function [36,37]. There is rising evidence that the lipid composition of the HDL particle is also important for HDL functions. Sphingosine-1-phosphate (S1P), a component of the HDL particle, has been recently demonstrated to be a cause of HDL dysfunction; low S1P levels induce HDL dysfunction, and S1P-loading the HDL particle corrects this HDL dysfunction [38,39]. The crucial role of S1P is underlined by a recently published study showing a significant reduction of oxidative stress by administrating S1P agonists in a porcine model of ischemia-reperfusion [40].

In agreement with previous analyses [14], the observed loss of anti-oxidant HDL function occurred independently of HDL cholesterol serum levels. HDL particles are suggested to be more relevant for cardioprotective functions of HDL than the absolute concentration of HDL cholesterol [21]. Analyses of clinical risk factors identified reduced kidney function as strongly associated with impaired anti-oxidant HDL function. This finding is in line with prior studies [41] and might be caused by higher inflammatory and oxidative stress levels in patients with reduced GFR [42]. Furthermore, our study identified a poor anti-oxidant HDL function in patients exposed to extracorporeal circulation (ECC), including renal replacement therapy as well as ECMO support. The underlying mechanisms remain unclear but a systemic stress response triggered by the artificial surface of ECC is known to induce excessive generation of ROS [43,44]. On the other hand, the ECC circuit may adsorb trace elements that are essential to the anti-oxidant response, specifically zinc and selenium [44,45]. Further subgroup analysis revealed significantly higher HOI at the first day of ICU admission compared to samples collected 48hours after ICU admission. Further analyses revealed that these differences in HOI were mainly influenced by the high HOI of surgical patients at ICU admission, which significantly improved within 48 hours. A strong inflammatory stress reaction with peak responses occurring 12 to 24hours after surgery might explain this observation [46].

In summary, the identification of impaired anti-oxidant HDL function as independent risk factor for poor clinical outcome complements the knowledge on risk stratification in critically ill patients. This suggests that anti-oxidant properties of HDL rather than total HDL serum levels should be considered for risk stratification. Interestingly, data on favorable effects of anti-oxidant therapies on clinical outcome in ICU patients are controversially discussed so far [7,8,9]. Results of the current study propose that restoration and maintenance of anti-oxidant HDL function might serve as a new additional therapeutic target in critically ill patients. Considering the persistent effect of impaired anti-oxidant HDL function on clinical outcome, high-risk patients might benefit from specific treatment not only during the ICU stay but also in close and long-term check-ups after hospital discharge. A recently published study suggests that statin therapy may improve anti-oxidant HDL function [13]. If future randomized clinical trials confirmed this finding, statins might become a promising therapeutic option in critically ill patients. In addition to medical treatment, intensified exercise training in ICU survivors might be a potential therapeutic approach, as exercise is known to have beneficial effects on HDL functions [47]. Whether early physical therapy interventions in the ICU might have favorable impact on protective functions of HDL has to be addressed by future studies.

A potential limitation of the present study is that the applied assay for determining the anti-oxidant HDL function has been performed with apolipoprotein B–depleted serum samples instead of using freshly isolated HDL. However, previous studies demonstrated that the use of apolipoprotein B–depleted serum samples provides valid and reproducible results as long as this technique is consistently applied for all measurements [48,49]. The patients under investigation represent a specific critically ill study population and it remains to be tested whether findings could be transferred to a more general patient population.

## Conclusion

We identified impaired anti-oxidant HDL capacity as a strong and independent predictor of short-term as well as long-term mortality in unselected critically ill patients. Reduced renal function and exposure to extracorporeal circulation were found associated with compromised anti-oxidant HDL function.
